# Diagnostic Accuracy of Combined PET/CT with MRI, 18F-FDG PET/MRI, and 18F-FDG PET/CT in Patients with Oropharyngeal and Hypopharyngeal Squamous Cell Carcinoma: A Systematic Review and Meta-Analysis

**DOI:** 10.1155/2021/6653117

**Published:** 2021-04-28

**Authors:** Mania Kave, Kambiz Sadegi, Fateme Parooie, Morteza Salarzaei

**Affiliations:** Zabol University of Medical Sciences, Zabol, Iran

## Abstract

**Introduction:**

The aim of this paper is to compare the diagnostic accuracy of PET/CT, PET/MRI, and the combination of PET/CT and MRI for detecting synchronous cancer and distant metastasis in patients with oropharyngeal and hypopharyngeal squamous cell carcinomas (OHSCC).

**Method:**

A large and growing body of literature has been conducted using the Preferred Reporting Items for Systematic Reviews (PRISMA). The researchers collected all accessible literature existing through Cochrane Library (John Wiley & Sons) electronic databases, Embase (Elsevier), PubMed (U.S. National Library of Medicine), Scopus, and Google Scholar up to June 2020. Analyses were conducted using Stata version 12.0 (StataCorp LP).

**Results:**

A total of nine studies consisting of 1166 patients were included. The pooled sensitivity of combined PET/CT with MRI, 18F-FDG PET/MRI, and 18F-FDG PET/CT was 0.92, 0.80, and 0.79, respectively, and the corresponding specificities were 0.93, 0.91, and 0.88. The overall prevalence of distant metastases and synchronous cancer in patients with oropharyngeal and hypopharyngeal squamous cell carcinomas was 9.2% and 11.8%, respectively, with the esophagus (4.6%) being the most common site of synchronous cancer. The most common sites of distant metastases were lung (3%), bone (1.2%), and distant lymph nodes (1.2%), respectively.

**Conclusion:**

Our study showed an approximately similar diagnostic performance for PET/CT, PET/MRI, and the combination of PET/CT and MRI for metastasis assessment in advanced oropharyngeal and hypopharyngeal squamous cell carcinomas.

## 1. Introduction

The common cancers of the head and neck include oropharyngeal and hypopharyngeal squamous cell carcinomas (OHSCC), which appear in adjacent anatomic areas. They usually have similar treatments, such as chemotherapy, radiotherapy, and surgery. However, the results of these treatments have not been satisfying, particularly in advanced cases [1]. Schaarschmidt et al. found that, in a period of five years, the overall survival (OS) was 59.7% and progression-free survival (PFS) was 40.7% [2]. There are some reasons for the high mortality of HSCC including early metastases, late presentations, and second primary cancers. Esophageal cancer was reported to be the most frequent synchronous malignancy in patients suffering from OHSCC [3–5]. It would be possible to assess the distant sites as well as the primary tumor areas with a single MRI examination [6, 7–9]. Moreover, it has been found that MRI is able to detect second primary tumors among patients suffering from untreated OHSCC (3). Another technical process that can integrate anatomic data from MRI and also metabolic information from PET is called 18F-FDG PET/MRI. So far, however, there is no evidence to prove any detrimental effect on the diagnostic performance, regarding integrating PET detectors with MRI scanners at the same time [10]. Studies show that there are similarities in diagnostic value of PET/MRI and PET/CT among HNC patients [11–14]. A considerable amount of published studies described patients with primary tumors emanating from different head and neck areas. Alternatively, there are no adequate studies on synchronous cancers or distant metastases. The aim of this paper is to conduct a systematic review and meta-analysis to determine the clinical capabilities of PET/MRI for detecting metastases among patients with OHSCC. Additionally, the performances of this novel technique are compared with those of PET/CT and the combination of MRI with PET/CT.

## 2. Material and Methods

### 2.1. Search Strategy

We searched for English articles in Cochrane Library, Medline, EMBASETM, Trip Database, and Google Scholar up to October 2020. The keywords used for searching process were (“Positron Emission Tomography” OR PET [MeSH] OR PET-CT) AND (“PET-MRI” OR “Positron Emission Tomography-MRI” OR “^18^F-FDG PET/MRI”) AND (“Magnetic Resonance Imaging” OR “MRI”) for the diagnostic test, and keywords included “oropharyngeal” OR “hypo-pharyngeal” OR “squamous cell carcinoma” OR “Head and neck SCC” OR “SCC” for the clinical region. The articles that were designed as reviews, letter to editors, comments, and case reports were excluded. In addition, we reviewed the references list of the included articles in order to find more relevant articles. Two reviewers independently evaluated the articles in terms of the inclusion criteria, and if there was a disagreement, it would be resolved by a discussion with the third reviewer.

### 2.2. Study Selection

A large and growing body of literature has been conducted using the Preferred Reporting Items for Systematic Reviews and Meta-Analysis (PRISMA). The researchers collected all accessible literature existing through Cochrane Library (John Wiley & Sons), electronic databases Embase (Elsevier), PubMed (U.S. National Library of Medicine), Scopus, and Google Scholar up to June 2020.

### 2.3. Inclusion and Exclusion Criteria

**Figure 1:**
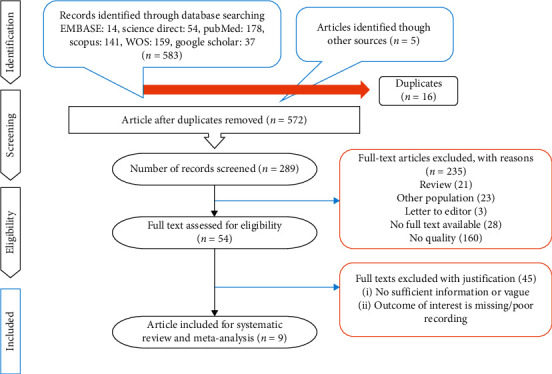
PRISMA flow diagram.

The present study addressed several factors, namely, (i) examining the performance of 18F-FDG PET/CT and PET MRI among those suffering from oropharyngeal and hypopharyngeal squamous cell carcinoma; (ii) imaging follow-up or histopathological analysis as the reference standard; (iii) expressing of stated values for false positive (FP), true positive (TN), true negative (TN), and false negative (FN). Studies were excluded if they (i) focused on prognosis or therapeutic response rather than detecting metastases; (ii) had less than 10 patients included; (iii) were published as a case report, conference abstract, review, letter, comment, or animal experiment; (iv) were not written in English. The researchers examined all full-text articles in the scope of inclusion criteria.  Figure 1 illustrates the PRISMA flowchart for the selection process.

### 2.4. Assessment of Methodological Quality

The Quality Assessment of Diagnostic Accuracy Studies (QUADAS-2) was examined by two reviewers to determine the eligibility criteria. This quality control instrument includes four subsections, including the selection of patients, tests that are considered as reference standard and index, and timing and flow of a study. Rating risks of bias was rated as low, high, or unclear. To be included in this study, researchers must have a low risk of bias. In the end, in case of any disagreement between reviewers, they reached a consensus considering the opinion of the third reviewer.

### 2.5. Data Extraction

Two reviewers analyzed the accepted literature by applying PRISMA guidelines. The data about year of publication, first author, country of studies, technical specifications, study design, and reference standard and FP, FN, TP, and TN were extracted from each article.

### 2.6. Statistical Analysis

To avoid clinical interstudy heterogeneity, studies that only possess PET MRI and 18F-FDG PET/CT evaluations simultaneously were included. In order to calculate the pooled sensitivities and specificities with 95% confidence intervals (95% CIs), the researchers used the bivariate random effects model of Reitsma et al. [15]. Moreover, the positive and negative likelihood ratios (PLRs and NLRs) and diagnostic odds ratios (DORs) were calculated. The result was considered significant at a two-tailed p-value of less than 0.10, by comparing the DORs of PET/MRI and PET/CT with using Z tests. The researchers transformed the natural logarithm of DOR in order to gain a normal distribution. By examining the curve, we drew the HSROCs, which stands for hierarchical summary receiver curves and then calculated the area under each curve. Applying the chi-square statistic for the pooled estimate, the heterogeneity among studies was assessed (*p* < 0.10 indicated a significant heterogeneity). The heterogeneity causes the variation across studies, which was estimated by calculating the *I*^2^ values. To assess for publication bias, Deeks' funnel plot was applied, as illustrated by an asymmetric appearance. Analyses were conducted using Stata version 12.0 (StataCorp LP).

## 3. Results

### 3.1. Literature Search

Our electronic searching process provided us with an overall 588 studies, from which 299 were excluded because they were duplicates or not written in English; we also excluded 235 studies, which included review, case report, and letters to editor. Finally, nine studies were included in this review (Figure 1).

### 3.2. Characteristics of the Included Studies

Nine studies consisting of 1166 patients with oropharyngeal and hypopharyngeal squamous cell carcinomas were included. The age range of the included population was 30–90 years, with a mean age range of 60.1 years. The male-to-female ratio was 6.7 (Table 1).

### 3.3. Methodological Quality

**Figure 2:**
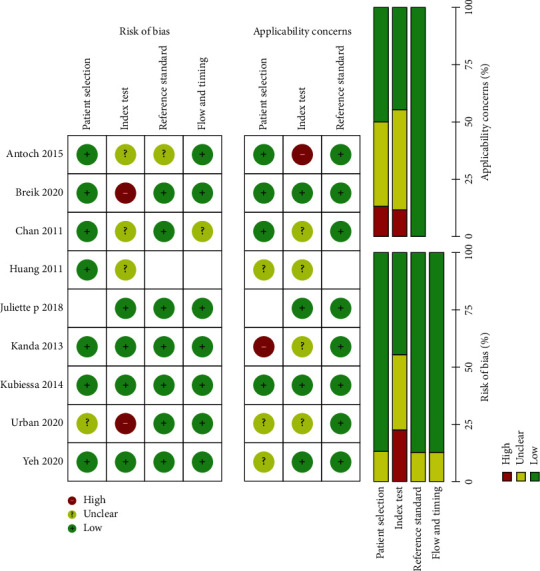
The risk of bias in the studies conducted was measured using QUADAS-2 tool. The risk of bias shown in equation 2 in the image model of each diagram indicates the number and percentage of studies with high (red), medium (yellow), and low (green) risk of bias in four groups of the QUADAS-2 tool.

We reported an overview of the QUADAS 2 scores in Figure 2. Overall, there was a moderate risk of bias due to index test. Regarding patients' selection, reference standard, and flow and timing of most studies, the risk of bias was low.

### 3.4. Heterogeneity between Studies and Publication Bias

The heterogenicity was significant for the nine included studies with respect to sensitivity and specificity for combined 18F-FDG PET/CT with MRI(*I*^2^ = 93.4%) and PET/CT(*I*^2^ = 72.7%). Heterogeneity was not statistically significant for PET/MRI (*I*^2^ = 57.4%) (Table 2).

### 3.5. Meta-Analysis of Diagnostic Accuracy of Combined MRI with PET/CT, PET MRI, and PET/CT

**Figure 3:**
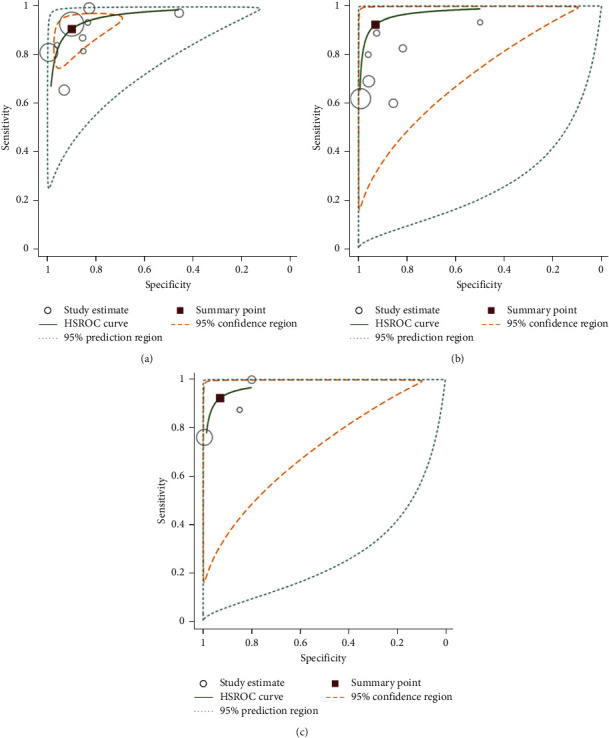
Hierarchical summary receiver (HSROC) curve for 18F-FDG PET/CT (a), 18F-FDG PET/MRI (b), and combined PET/CT with MRI (c) for the detection of distant metastases in patients with oropharyngeal and hypopharyngeal squamous cell carcinomas.

**Figure 4:**
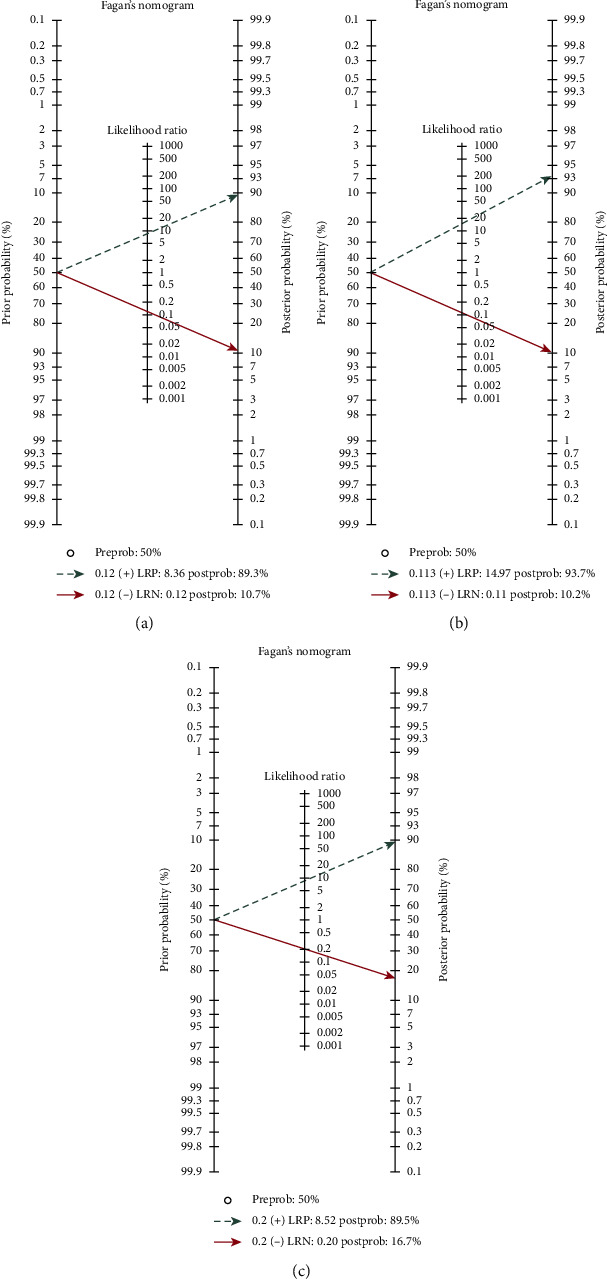
Fagan's nomogram for the calculation of posttest probabilities.

**Figure 5:**
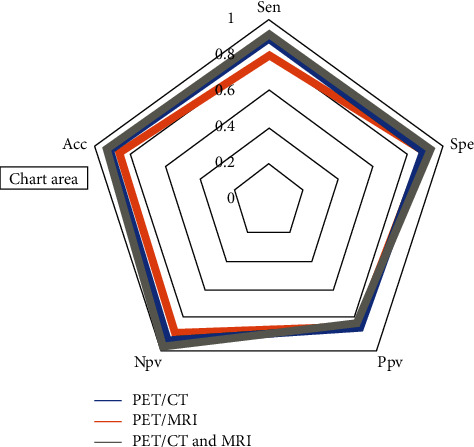
Radar chart for comparison of combined PET/CT with MRI, 18F-FDG PET/MRI, and 18F-FDG PET/CT for the detection of synchronous cancers and distant metastases in patients with oropharyngeal and hypopharyngeal squamous cell carcinomas.

The pooled results showed that the sensitivity and specificity of combined MRI with PET/CT were 0.92 (95% CI 76%–97%) and 0.93 (95% CI 74%–98%), respectively, and the DOR and accuracy for this combined method were 160.52 (95% CI 44.81–575) and 0.94 (95% CI 92%–97%), respectively. PET/CT alone showed a sensitivity of 0.79 (95% CI 72%–85%), specificity of 0.88 (95% CI 83%–92%), and accuracy of 0.87 (95% CI 83%–91%). The DOR for PET/CT was 85.51 (95% CI 39.43–185.46). The pooled sensitivity, specificity, DOR, and accuracy for PET/MRI alone in detection of distant metastases in patients with oropharyngeal and hypopharyngeal squamous cell carcinomas were 0.80 (95% CI 69%–87%), 0.91 (95% CI 79%–96%), 44.41 (95% CI 20.17–97.78), and 0.92(95% CI 90%–93%), respectively (Table 2; Figure 3). The overall areas under the curve for combined MRI with PET/CT, PET MRI, and PET/CT using a hierarchical summary receiver (HSROC) curve were 0.9790, 0.9154, and 0.9555, respectively (Figure 4). The most useful values are considered to be negative and positive predictive values (NPV and PPV). However, these values depend on factors such as disease prevalence. The PPV for combined MRI with PET/CT, PET MRI, and PET/CT were 0.82, 0.84, and 0.85, respectively, and the corresponding NPV for these methods were 0.99, 0.89, and 0.95, respectively.  Figure 5 shows the Fagan's nomogram. A pretest probability of 50% for all three diagnostic tools was fixed, which was estimated by the number of symptomatic cases in selected studies. PET CT had a posttest probability of 89.3%. For PET MRI (b), the posttest probability was 93.1%, and combined PET CT with MRI (c) had a posttest probability of 89.5%. If a patient's test was positive, the posttest probability that the patient truly had developed metastases would be 10.7% (a) or 10.2 (b) or 16.7 (c) (solid line in red). The results were obtained using the following calculations: pretest odds = prevalence/1—prevalence; posttest odds = pretest odds × LR – (LR+); posttest probability = posttest odds/1 + posttest odds (Figure 5).

The reported sensitivity of combined MRI with PET/CT ranged from 76% in Taiwan (based on three included studies) and 100% in Netherlands (based on one study included), while the corresponding specificities were 80% and 99%.

**Figure 6:**
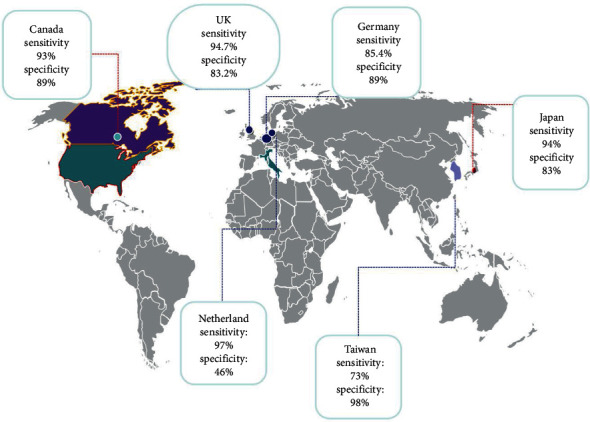
Sensitivity and specificity of 18F-FDG PET/CT for the detection of distant metastases in patients with oropharyngeal and hypopharyngeal squamous cell carcinomas based on the countries.

For PET/MRI, the highest sensitivity and specificity belonged to Japan (sensitivity: 94%, specificity: 83%) based on one included study, and PET/CT was reported to have the highest sensitivity and specificity in Netherlands (97%) and Taiwan (98%), respectively (Figure 6).

### 3.6. Meta-Analysis of the Prevalence of Distant Metastases and Synchronous Cancer in Patients with Oropharyngeal and Hypopharyngeal Squamous Cell Carcinoma

The overall prevalence of distant metastases and synchronous cancer in patients with oropharyngeal and hypopharyngeal squamous cell carcinomas were 9.2% (95% CI: 6, 12.5) and 11.8% (95% CI: 8.2, 15.5), respectively, with the esophagus (4.6%) being the most common site of synchronous cancer. The most common sites of distant metastases were lung (3%), bone (1.2%), and distant lymph nodes (1.2%).

## 4. Discussion

In the present study, the researchers presented a higher sensitivity of PET/CT combined with MRI compared with PET/MRI or PET/CT alone in the diagnosis of distant metastases in patients with oropharyngeal and hypopharyngeal squamous cell carcinomas. There is a large volume of published studies indicating that the role of PET/MRI is generally the same as the diagnostic ability of PET/CT in the head and the neck region [24–26]. The advantages of CT for the evaluation of head and neck lesions are the easy accessibility and having a fewer motion artifacts. However, sometimes additional MRI is recommended for cases with previously obtained PET/CT. This is for the simple reason that MRI is proved to perform better in soft-tissue contrast in structures that are complex. MRI also is preferred because of its better depiction of the cancerous bone marrow of craniofacial bones. Moreover, the fact that MRI does not expose the patient to any kind of radiation and has no limitation for patients with dental prostheses must be considered [26–29]. The generalizability of much published research on this issue has shown that the accuracy of MRI in detecting second primary cancers or distant metastases is comparable to that of PET/CT in HNC [18, 30]. The large body of previous studies is in accordance with the present systematic review suggesting that MRI and PET/CT have various superiorities for diagnosis of malignancies in different sites. Because liver and brain lesions have high FDG uptake physiologically and small lesions may be obscured, MRI can be more accurate in detecting these lesions [31]. However, using MRI sometimes does not lead to the correct result when detecting lesions in the lungs and intestines and distant nodes [31, 32]. Catalano et al., in their meta-analysis, pointed that PET/MRI outperformed PET/CT and was more sensitive in detection of metastases of liver and bone. They also reported that PET/MRI was more accurate in assessing lymphadenopathy and local staging of pelvic malignancies [33]. In this paper, the most sensitive results were obtained through the combination of PET/CT and MRI with a sensitivity of 92% and a specificity of 93%. Some known MRI deficiencies are compensated by PET. While CT scans can also fill in some of the remaining MRI gaps, these two factors make combined PET/CT and MRI more sensitive compared with using PET/MRI or PET/CT only. The results of the present study indicated a sensitivity of 80% and a specificity of 91% for PET/MRI, while the corresponding ratios for PET/CT were 79.4% and 88.2%. However, as shown in HSROC curve, confidence intervals for PET/CT combined with MRI, PET/CT, and PET/MRI were overlapping, so no significant difference in terms of the diagnostic performance was found between these three methods. Partovi et al. concluded in their study that imaging of lymph nodes and distant metastases in patients with head and neck cancer is the same on both 18F-FDG-PET/MR and PET/CT [34]. Broadly speaking, CT is predicted to be suitable for assessing lesions located in sites such as lungs, while MRI is better for diagnosing head and neck and intracranial lesions. Another associated factor causing these differences can be different acquisition protocols. The prevalence of distant metastases varies widely according to the published data, with an estimated 18.2% prevalence for the previous studies and 2.8% to 23.8% for the new ones [35–38]. A possible explanation for this different statistic is the classification of patients with head and neck tumors of different areas in one group. From the previously mentioned data, the prevalence of synchronous cancers/distant metastases seems to be more in patients with oropharyngeal and hypopharyngeal squamous cell carcinomas compared with patients with oral cavity cancers [39]. The overall prevalence of distant metastases and synchronous cancer in patients with oropharyngeal and hypopharyngeal squamous cell carcinomas was 9.2% (95% CI: 6, 12.5) and 11.8% (95% CI: 8.2, 15.5), respectively, with the esophagus (4.6%) being the most common site of synchronous cancer. The most common sites of distant metastases were lung (3%), bone (1.2%), and distant lymph nodes (1.2%). The meta-analysis presented in this study has different limitations. First, some parameters of the scan in different studies may affect the accuracy of MRI and PET/CT, which were not considered in this study due to lack of data. Second, the design of most of the included studies was retrospective and did not use a blinded method. Third, the small number of articles included in this study may lead to bias.

## 5. Conclusion

Our study showed an approximately similar diagnostic performance for PET/CT, PET/MRI, and the combination of PET/CT and MRI for metastasis assessment in oropharyngeal and hypopharyngeal squamous cell carcinomas.

## Figures and Tables

**Table 1:** Characteristics of the included studies.

Author	Country	Year	Number of patients	Number of males and females	Age mean ± SD or range	Duration	Study design
Driessen et al. [16]	Netherlands	2018	70	51/19	61 (42–81)	2011–2014	Prospective
Breik et al. [17]	UK	2020	140	111/29	68	2010–2017	Retrospective
Chan et al. [18]	Taiwan	2011	103	97/6	53.6 ± 9	2006–2008	Prospective
Yeh et al. [19]	Taiwan	2020	198	187/11	56.0 ± 9.8	—	Prospective
Huang et al. [20]	Taiwan	2011	27	26/1	—	2008/2010	Prospective
Schaarschmidt et al. [2]	Germany	2015	25	23/2	56.5 ± 7.7		Prospective
Kubiessa et al. [21]	Germany	2014	17	17/4	60 (42–78)	—	Prospective
Urban et al. [22]	Canada	2020	556	482/74	60 (33–90)	2012–2016	Retrospective
Kanda et al. [23]	Japan	2013	30	24/6	66.9 ± 11.1	2011–2013	Prospective

**Table 2:** Diagnostic performance of combined MRI with PET/CT, PET MRI, and PET/CT in patients with synchronous cancers and distant metastases in patients with oropharyngeal and hypopharyngeal squamous cell carcinomas.

Parameter	PET/CT	PET/MRI	Combined PET/CT with MRI
Sensitivity (95% CI)	0.79 (95% CI 72%–85%)	0.80 (95% CI 69%–87%)	0.92 (95% CI 76%–97%)
Specificity (95% CI)	0.88(95% CI 83%–92%)	0.91 (95% CI 79%–96%)	0.93 (95% CI 74%–98%)
PLR (95% CI)	8.95 (95% CI 4.21–19.03)	9.64 (95% CI 3.93–23.64)	13.22 (95% CI 3.36–52.06)
NLR (95% CI)	0.10 (95% CI 0.05–0.19)	0.21 (95% CI 0.14–0.32)	0.08 (95% CI 0.02–0.26)
DOR (95% CI)	85.51 (95% CI 39.43–185.46)	44.41 (95% CI 20.17–97.78)	160.52 (95% CI 44.81–575)
PPV	0.85	0.84	0.82
NPV	0.95	0.89	0.99
Accuracy	0.87 (95% CI 83%–91%)	0.92 (95% CI 90%–93%)	0.94 (95% CI 92%–97%)
AUC-SROC			
*I* ^2^	72.7%	57.4%	93.4%
Q index	7.15	7.32	30.11

## Data Availability

The data used in this study are available upon reasonable request from the corresponding author.

## References

[1] Szyszko T. A., Cook G. J. R. (2018). PET/CT and PET/MRI in head and neck malignancy. *Clinical Radiology*.

[2] Schaarschmidt B. M., Heusch P., Buchbender C. (2016). Locoregional tumour evaluation of squamous cell carcinoma in the head and neck area: a comparison between MRI, PET/CT and integrated PET/MRI. *European Journal of Nuclear Medicine and Molecular Imaging*.

[3] Rohde M., Nielsen A. L., Johansen J. (2018). Up-front PET/CT changes treatment intent in patients with head and neck squamous cell carcinoma. *European Journal of Nuclear Medicine and Molecular Imaging*.

[4] Ng S.-H., Liao C.-T., Lin C.-Y. (2016). Dynamic contrast-enhanced MRI, diffusion-weighted MRI and 18F-FDG PET/CT for the prediction of survival in oropharyngeal or hypopharyngeal squamous cell carcinoma treated with chemoradiation. *European Radiology*.

[5] Kuno H., Qureshi M. M., Chapman M. N. (2017). CT texture analysis potentially predicts local failure in head and neck squamous cell carcinoma treated with chemoradiotherapy. *American Journal of Neuroradiology*.

[6] Wong K. H., Panek R., Welsh L. (2016). The predictive value of early assessment after 1 cycle of induction chemotherapy with 18F-FDG PET/CT and diffusion-weighted MRI for response to radical chemoradiotherapy in head and neck squamous cell carcinoma. *Journal of Nuclear Medicine*.

[7] Wong C. K., Chan S. C., Ng S. H. (2019). Textural features on 18F-FDG PET/CT and dynamic contrast-enhanced MR imaging for predicting treatment response and survival of patients with hypopharyngeal carcinoma. *Medicine*.

[8] Suenaga Y., Kitajima K., Kanda T. (2016). [18F]-FDG PET/CT imaging for detection of nodal metastases in patients with squamous cell carcinoma of the pharynx and larynx: comparison with CT. *Japanese Journal of Radiology*.

[9] Liu Y. (2019). FDG PET/CT for metastatic squamous cell carcinoma of unknown primary of the head and neck. *Oral Oncology*.

[10] Bahig H., Lapointe A., Bedwani S. (2019). Dual-energy computed tomography for prediction of loco-regional recurrence after radiotherapy in larynx and hypopharynx squamous cell carcinoma. *European Journal of Radiology*.

[11] Wegner I., Hooft L., Reitsma J. B. (2016). MRI versus CT versus 18F‐FDG PET‐CT for detecting lymph node metastases in patients with head and neck squamous cell carcinoma. *The Cochrane Database of Systematic Reviews*.

[12] Reitsma J. B., Glas A. S., Rutjes A. W. S., Scholten R. J. P. M., Bossuyt P. M., Zwinderman A. H. (2005). Bivariate analysis of sensitivity and specificity produces informative summary measures in diagnostic reviews. *Journal of Clinical Epidemiology*.

[13] Driessen J. P., Peltenburg B., Philippens M. E. P. (2019). Prospective comparative study of MRI including diffusion-weighted images versus FDG PET-CT for the detection of recurrent head and neck squamous cell carcinomas after (chemo)radiotherapy. *European Journal of Radiology*.

[14] Breik O., Kumar A., Birchall J., Mortimore S., Laugharne D., Jones K. (2020). Follow up imaging of oral, oropharyngeal and hypopharyngeal cancer patients: comparison of PET-CT and MRI post treatment. *Journal of Cranio-Maxillofacial Surgery*.

[15] Chan S.-C., Wang H.-M., Yen T.-C. (2011). 18F-FDG PET/CT and 3.0-T whole-body MRI for the detection of distant metastases and second primary tumours in patients with untreated oropharyngeal/hypopharyngeal carcinoma: a comparative study. *European Journal of Nuclear Medicine and Molecular Imaging*.

[16] Yeh C.-H., Chan S.-C., Lin C.-Y. (2020). Comparison of 18F-FDG PET/MRI, MRI, and 18F-FDG PET/CT for the detection of synchronous cancers and distant metastases in patients with oropharyngeal and hypopharyngeal squamous cell carcinoma. *European Journal of Nuclear Medicine and Molecular Imaging*.

[17] Huang S.-H., Chien C.-Y., Lin W.-C. (2011). A comparative study of fused FDG PET/MRI, PET/CT, MRI, and CT imaging for assessing surrounding tissue invasion of advanced buccal squamous cell carcinoma. *Clinical Nuclear Medicine*.

[18] Kubiessa K., Purz S., Gawlitza M. (2014). Initial clinical results of simultaneous 18F-FDG PET/MRI in comparison to 18F-FDG PET/CT in patients with head and neck cancer. *European Journal of Nuclear Medicine and Molecular Imaging*.

[19] Urban R., Godoy T., Olson R. (2020). FDG-PET/CT scan assessment of response 12 weeks post radical radiotherapy in oropharynx head and neck cancer: the impact of p16 status. *Radiotherapy and Oncology*.

[20] Kanda T., Kitajima K., Suenaga Y. (2013). Value of retrospective image fusion of 18F-FDG PET and MRI for preoperative staging of head and neck cancer: comparison with PET/CT and contrast-enhanced neck MRI. *European Journal of Radiology*.

[21] Sekine T., de Galiza Barbosa F., Kuhn F. P. (2017). PET + MR versus PET/CT in the initial staging of head and neck cancer, using a trimodality PET/CT + MR system. *Clinical Imaging*.

[22] Castaldi P., Leccisotti L., Bussu F., Micciche F., Rufini V. (2013). Role of 18F-FDG PET-CT in head and neck squamous cell carcinoma. *ACTA Otorhinolaryngologica Italica*.

[23] Spick C., Herrmann K., Czernin J. (2016). 18F-FDG PET/CT and PET/MRI perform equally well in cancer: evidence from studies on more than 2,300 patients. *Journal of Nuclear Medicine*.

[24] Queiroz M. A., Huellner M. W. (2015). PET/MR in cancers of the head and neck. *Seminars in Nuclear Medicine*.

[25] Kuhn F. P., Hüllner M., Mader C. E. (2014). Contrast-enhanced PET/MR imaging versus contrast-enhanced PET/CT in head and neck cancer: how much MR information is needed?. *Journal of Nuclear Medicine*.

[26] Ladefoged C. N., Hansen A. E., Keller S. H. (2015). Dental artifacts in the head and neck region: implications for dixon-based attenuation correction in PET/MR. *EJNMMI Physics*.

[27] Chan S.-C., Yeh C.-H., Yen T.-C. (2018). Clinical utility of simultaneous whole-body 18F-FDG PET/MRI as a single-step imaging modality in the staging of primary nasopharyngeal carcinoma. *European Journal of Nuclear Medicine and Molecular Imaging*.

[28] Yi C. A., Shin K. M., Lee K. S. (2008). Non-small cell lung cancer staging: efficacy comparison of integrated PET/CT versus 3.0-T whole-body MR imaging. *Radiology*.

[29] Schmidt G. P., Haug A. R., Schoenberg S. O., Reiser M. F. (2006). Whole-body MRI and PET-CT in the management of cancer patients. *European Radiology*.

[30] Catalano O. A., Rosen B. R., Sahani D. V. (2013). Clinical impact of PET/MR imaging in patients with cancer undergoing same-day PET/CT: initial experience in 134 patients-A hypothesis-generating exploratory study. *Radiology*.

[31] Partovi S., Kohan A., Vercher-Conejero J. L. (2014). Qualitative and quantitative performance of18F-FDG-PET/MRI versus18F-FDG-PET/CT in patients with head and neck cancer. *American Journal of Neuroradiology*.

[32] Krishnatreya M., Rahman T., Kataki A., Das A., Das A., Lahkar K. (2013). Synchronous primary cancers of the head and neck region and upper aero digestive tract: defining high-risk patients. *Indian Journal of Cancer*.

[33] Wang Y.-K., Chuang Y.-S., Wu T.-S. (2017). Endoscopic screening for synchronous esophageal neoplasia among patients with incident head and neck cancer: prevalence, risk factors, and outcomes. *International Journal of Cancer*.

[34] Li X., Di B., Shang Y., Zhou Y., Cheng J., He Z. (2009). Clinicopathologic risk factors for distant metastases from head and neck squamous cell carcinomas. *European Journal of Surgical Oncology (EJSO)*.

[35] Kuperman D. I., Auethavekiat V., Adkins D. R. (2011). Squamous cell cancer of the head and neck with distant metastasis at presentation. *Head & Neck*.

[36] Kim Y., Roh J.-L., Kim J. S. (2019). Chest radiography or chest CT plus head and neck CT versus 18F-FDG PET/CT for detection of distant metastasis and synchronous cancer in patients with head and neck cancer. *Oral Oncology*.

[37] Park J. T., Roh J.-L., Kim J. S. (2016). 18F FDG PET/CT versus CT/MR imaging and the prognostic value of contralateral neck metastases in patients with head and neck squamous cell carcinoma. *Radiology*.

[38] Suenaga Y., Kitajima K., Ishihara T. (2016). FDG-PET/contrast-enhanced CT as a post-treatment tool in head and neck squamous cell carcinoma: comparison with FDG-PET/non-contrast-enhanced CT and contrast-enhanced CT. *European Radiology*.

[39] Toya R., Saito T., Matsuyama T. (2020). Diagnostic value of FDG-PET/CT for the identification of extranodal extension in patients with head and neck squamous cell carcinoma. *Anticancer Research*.

